# Supplementary material for: CYP6AE gene cluster knockout in Helicoverpa armigera reveals role in detoxification of phytochemicals and insecticides
Source: Nat Commun. 2018 Nov 16;9:4820. doi: 10.1038/s41467-018-07226-6 (PMC6240031; doi:10.1038/s41467-018-07226-6)
Supplement: Supplementary file 1 — Supplementary Information [file 41467_2018_7226_MOESM1_ESM.pdf]

Supplementary Information

CYP6AE gene cluster knockout in *Helicoverpa armigera* reveals role in detoxification of phytochemicals and insecticides.

Wang et al.

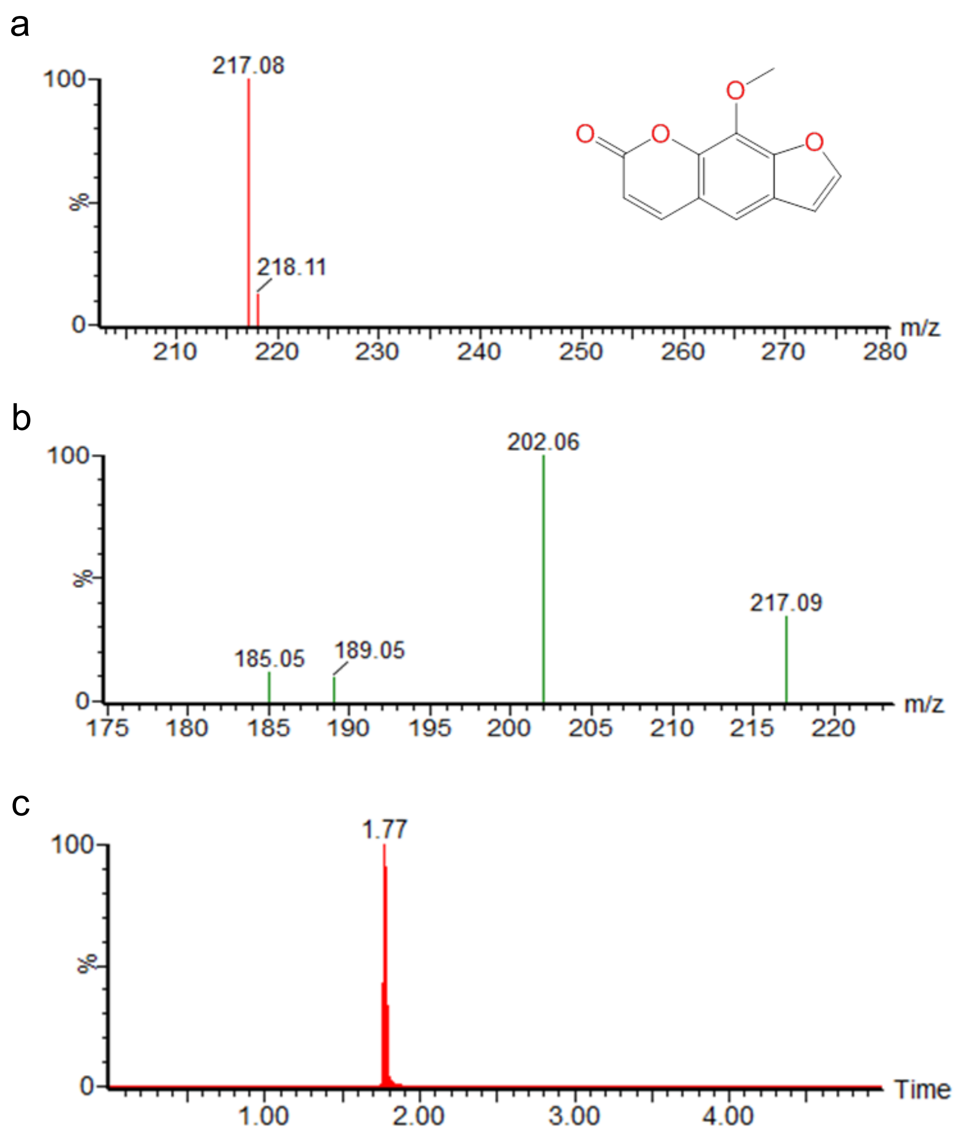

Supplementary Figure 1. Detection of xanthotoxin by UPLC-MS/MS. **a.** Full scan of xanthotoxin. **b.** Fragments found in MS/MS spectrum. **c.** MRM spectrum of xanthotoxin. Xanthotoxin eluted at 1.77min.

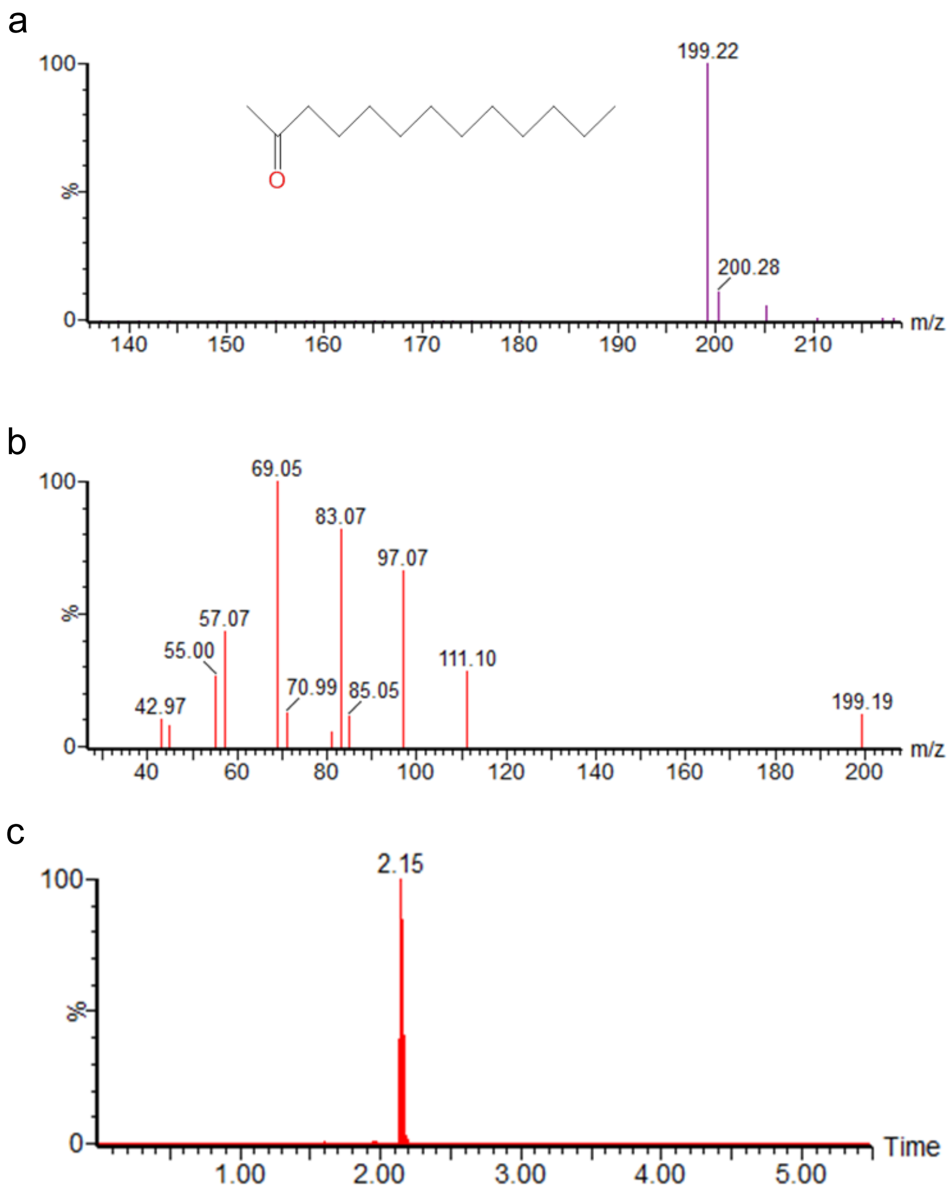

Supplementary Figure 2. Detection of 2-tridecanone by UPLC-MS/MS. **a.** Full scan of 2-tridecanone. **b.** Fragments found in MS/MS spectrum. **c.** MRM spectrum of 2-tridecanone. 2-tridecanone eluted at 2.15min.

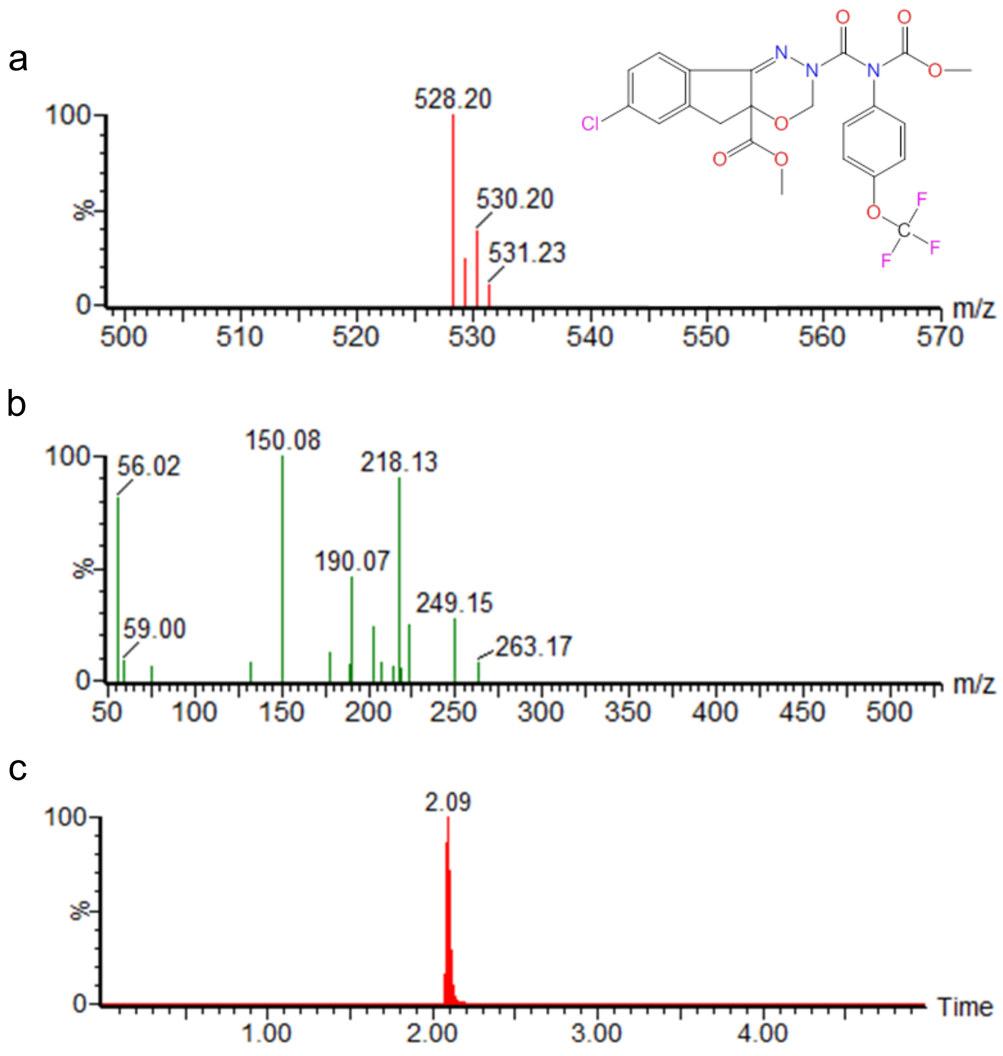

Supplementary Figure 3. Detection of indoxacarb by UPLC-MS/MS. **a.** Full scan of indoxacarb. **b.** Fragments found in MS/MS spectrum. **c.** MRM spectrum of indoxacarb. Indoxacarb eluted at 2.09min.

Supplementary Table 1

Susceptibility of newly-hatched larvae of the unedited (SCD) and CYP6AE knockout strains (SCD-d6AE) of *H. armigera* to diverse phytochemicals.

Plant allelochemical	Strain	LC_{50} (mg/g)	95% FL (mg/g)	Slope±SE	Toxicity ratio
Xanthotoxin	SCD	7.00	4.94-9.32	3.24±0.35	4.1
	SCD-d6AE	1.73	1.50-1.92	5.87±0.86	
Gossypol acetate	SCD	5.30	4.33-6.49	4.13±0.93	1.2
	SCD-d6AE	4.31	2.25-6.02	3.68±0.89	
2-Tridecanone	SCD	3.28	2.87-3.62	6.78±1.18	1.4
	SCD-d6AE	2.38	1.91-2.69	6.61±1.36	
Nicotine	SCD	1.64	1.36-1.93	9.26±2.10	1.0
	SCD-d6AE	1.60	1.24-1.93	8.46±2.24	
Coumarin	SCD	1.87	1.54-2.24	4.71±0.84	1.1
	SCD-d6AE	1.65	0.90-2.08	5.37±1.75	

LC_{50}, lethal concentration that kills 50% of *H. armigera* larvae; 95% FL, 95% fiducial limits of LC_{50}; Toxicity ratio was calculated as LC_{50} of SCD/LC_{50} of SCD-d6AE; LC_{50} values were considered significantly different if their fiducial limits did not overlap[1].

Supplementary Table 2

Susceptibility of second instar larvae of the unedited (SCD) and CYP6AE knockout strains (SCD-d6AE) of *H. armigera* to diverse insecticides.

Insecticide	Strain	LC_{50} (mg/L)	95% FL (mg/L)	Slope±SE	Toxicity ratio
Esfenvalerate	SCD	5.13	3.97-6.23	2.85±0.43	4.5
	SCD-d6AE	1.14	0.82-1.64	1.80±0.16	
Indoxacarb	SCD	5.12	4.52-5.84	2.91±0.29	3.1
	SCD-d6AE	1.64	1.84-2.34	2.35±0.26	
Emamectin benzoate	SCD	0.015	0.010-0.022	5.47±0.68	1.0
	SCD-d6AE	0.015	0.013-0.016	9.81±1.70	
Chlorantraniliprole	SCD	1.20	0.87-1.67	1.47±0.21	0.7
	SCD-d6AE	1.83	1.26-2.55	1.87±0.38	

LC_{50}, lethal concentration that kills 50% of *H. armigera* larvae; 95% FL, 95% fiducial limits of LC_{50}; Toxicity ratio was calculated as LC_{50} of SCD/LC_{50} of SCD-d6AE; LC_{50} values were considered significantly different if their fiducial limits did not overlap[1].

Supplementary Table 3
Metabolism of xanthotoxin, 2-tridecanone and indoxacarb by CYP6AE P450s.

P450	*V* (pmol substrate/min/pmol P450)		
	Xanthotoxin	2-Tridecanone	Indoxacarb
CYP6AE11	n.d.	0.56±0.08	n.d.
CYP6AE14	n.d.	0.34±0.04	n.d.
CYP6AE16	n.d.	n.d.	n.d.
CYP6AE17	n.d.	n.d.	0.21±0.03
CYP6AE18	n.d.	n.d.	0.11±0.01
CYP6AE12	n.d.	n.d.	n.d.
CYP6AE15	n.d.	n.d.	n.d.
CYP6AE24	n.d.	n.d.	n.d.
CYP6AE19	3.32±0.43	0.56±0.07	n.d.
CYP6AE20	n.d.	n.d.	n.d.
CYP6B1	12.71±0.23	n.d.	-

"n.d." means the activity was not detected (limits of detection were 0.081, 0.033 and 0.016 pmol/min/pmol P450 for xanthotoxin, 2-tridecanone and indoxacarb, respectively). "-" means the activity was not tested.

Supplementary Table 4

Quantitative real-time PCR analysis of expression of CYP6AE subfamily genes in the larval midgut and fat body of *H. armigera* SCD strain. Gene expression values are indicated as expression folds in the larvae exposed to each xenobiotic compared with unexposed larvae (control).

Tissue	P450 name	Control	Xanthotoxin	2-Tridecanone	Gossypol	Esfenvalerate
Midgut	CYP6AE11	1.00±0.24	0.19±0.03	1.67±0.35	0.65±0.16	0.94±0.37
	CYP6AE14	1.00±0.21	5.28±1.52	2.85±0.57	40.47±9.36	5.06±1.97
	CYP6AE16	1.00±0.09	0.33±0.04	0.65±0.09	0.35±0.04	0.83±0.16
	CYP6AE17	1.00±0.09	0.67±0.06	1.57±0.35	0.53±0.11	1.01±0.23
	CYP6AE18	1.00±0.37	15.70±4.77	2.38±1.30	1.00±0.10	0.73±0.10
	CYP6AE12	1.00±0.16	0.93±0.19	1.14±0.14	1.63±0.37	2.32±0.39
	CYP6AE15	1.00±0.19	0.10±0.04	0.49±0.23	0.40±0.08	1.10±0.39
	CYP6AE24	1.00±0.16	0.61±0.22	1.21±0.62	2.47±0.80	0.39±0.09
	CYP6AE19	1.00±0.20	7836±448	3.11±0.32	2.34±0.61	8.75±3.80
	CYP6AE20	1.00±0.09	14523±6650	0.73±0.13	0.83±0.07	1.00±0.12
Fat body	CYP6AE11	1.00±0.29	3.08±0.35	2.02±1.58	0.40±0.24	0.36±0.17
	CYP6AE14	0.00	0.00	0.00	0.00	0.00
	CYP6AE16	1.00±0.13	0.72±0.10	0.57±0.08	0.64±0.11	0.46±0.08
	CYP6AE17	1.00±0.15	1.28±0.18	1.05±0.24	1.53±0.23	0.86±0.21
	CYP6AE18	1.00±0.27	1.04±0.60	1.72±0.97	2.67±1.14	2.10±1.07
	CYP6AE12	1.00±0.12	0.46±0.07	0.74±0.11	0.83±0.13	0.69±0.06
	CYP6AE15	1.00±0.09	4.84±1.29	1.35±0.58	2.21±0.75	1.31±0.37
	CYP6AE24	1.00±0.20	0.65±0.25	0.80±0.18	0.65±0.14	1.01±0.32
	CYP6AE19	1.00±0.26	250±14	0.81±0.11	2.21±0.40	1.45±0.52
	CYP6AE20	1.00±0.06	173±55	0.99±0.27	0.64±0.09	0.76±0.14

Supplementary Table 5
PCR primers used in this study.

Purpose	Primer name	Sequence (5'-3')
Preparation of template DNAs for sgRNAs	sgCYP6AE14F	TAATACGACTCACTATAGTTTCCCAACGTACCATACG
	sgCYP6AE14R	TTCTAGCTCTAAAACCGTATGGTACGTTGGGAAAC
	sgCYP6AE12F	TAATACGACTCACTATAGGATCTTGGACGATGAGCGC
	sgCYP6AE12R	TTCTAGCTCTAAAACGCGCTCATCGTCCAAGATCC
Detection of the CYP6AE cluster deletion	14F	AAACCTTTCCCTGTCGGCAT
	12R	AGATGGCCAAATAGCCCGAG
	20f	ACACCATGGCACCAATCCAT
	20r	CAGATGTCTCGTAACCCGCA
	15f	GGCATTTTTAGCGCACGTCA
	15r	TTGCTAGCACTGGGTACAGC
	12f	CCGCATTTGAAACCAGTCCC
	12r	AGCCCGAGCAACATTCTTGA
RT-qPCR analysis [2]	CYP6AE11-F	AAAAGTTCCCCAAAGCCCCA
	CYP6AE11-R	CTCACGGCCACTGAAGAAGT
	CYP6AE12-F	ACCCAGTACTAGGGCTCCTG
	CYP6AE12-R	TAGGACCTTCACCAAACGGC
	CYP6AE14-F	ATGTTAAGCCGGGTCCAAGG
	CYP6AE14-R	AAGTGGTGGCCGAAGTTTCA
	CYP6AE15-F	GAATCAGCGGCGAAAGTGAC
	CYP6AE15-R	TTGCTAGCACTGGGTACAGC
	CYP6AE16-F	GACAAATTCCCGAACGCACC
	CYP6AE16-R	TGAACTCCGCTACTTCACGG
	CYP6AE17-F	GACCGTACACCTACATGCCC
	CYP6AE17-R	TGGCGTGAACTCCACTTTTG
	CYP6AE18-F	CAGACCATACACCTACATGCCA
	CYP6AE18-R	CCGAGAAGTCTACGGTCGTG
	CYP6AE19-F	TGTGCCGTACCACAAACCTT
	CYP6AE19-R	CCGCAGTAGTCTGTCGACTC
	CYP6AE20-F	CGCTTTTGGGCAATTACGCT
	CYP6AE20-R	CCATCGGTCTCCTGCATTGA
	CYP6AE24-F	GGGGTCGACTGCTCAAGAAA
	CYP6AE24-R	GCAGCAAAGAACACGAAGCA

Ha-actin-F	CCTGGTATTGCTGACCGTATGC
Ha-actin-R	CTGTTGGAAGGTGGAGAGGGAA
EF-1α-F	GACAAACGTACCATCGAGAAG
EF-1α-R	GATACCAGCCTCGAACTCAC

Supplementary Table 6
Analysis parameters for substrate detection.

Substrates	Parent ion (m/z)	Daughter ions (m/z)	Cone voltage (V)	Collision energy (V)
Xanthotoxin	217	202*<br>189	30	20
2-Tridecanone	199.2	69*<br>83	30	10
Indoxacarb	528.2	150.1*<br>218.1	25	23

* quantitative daughter ions.

Supplementary References

1. Payton ME, Greenstone MH, Schenker, N (2003) Overlapping confidence intervals or standard error intervals: what do they mean in terms of statistical significance? *J Insect Sci* 3:34.

2. Shi Y, et al. (2018) Phylogenetic and functional characterization of ten P450 genes from the CYP6AE subfamily of *Helicoverpa armigera* involved in xenobiotic metabolism. *Insect Biochem Mol Biol* 93:79-91.
