# Supplementary material for: CYP6AE gene cluster knockout in Helicoverpa armigera reveals role in detoxification of phytochemicals and insecticides
Source: Nat Commun. 2018 Nov 16;9:4820. doi: 10.1038/s41467-018-07226-6 (PMC6240031; doi:10.1038/s41467-018-07226-6)
Supplement: Supplementary file 3 — Reporting Summary [file 41467_2018_7226_MOESM3_ESM.pdf]

nature research

Corresponding author(s): Yidong Wu

Reporting Summary

Nature Research wishes to improve the reproducibility of the work that we publish. This form provides structure for consistency and transparency in reporting. For further information on Nature Research policies, see Authors & Referees and the Editorial Policy Checklist.

Statistical parameters

When statistical analyses are reported, confirm that the following items are present in the relevant location (e.g. figure legend, table legend, main text, or Methods section).

n/a	Confirmed	
☐	☒	The exact sample size (*n*) for each experimental group/condition, given as a discrete number and unit of measurement
☐	☒	An indication of whether measurements were taken from distinct samples or whether the same sample was measured repeatedly
☐	☒	The statistical test(s) used AND whether they are one- or two-sided<br>*Only common tests should be described solely by name; describe more complex techniques in the Methods section.*
☒	☐	A description of all covariates tested
☒	☐	A description of any assumptions or corrections, such as tests of normality and adjustment for multiple comparisons
☐	☒	A full description of the statistics including central tendency (e.g. means) or other basic estimates (e.g. regression coefficient) AND variation (e.g. standard deviation) or associated estimates of uncertainty (e.g. confidence intervals)
☐	☒	For null hypothesis testing, the test statistic (e.g. *F*, *t*, *r*) with confidence intervals, effect sizes, degrees of freedom and *P* value noted<br>*Give P values as exact values whenever suitable.*
☒	☐	For Bayesian analysis, information on the choice of priors and Markov chain Monte Carlo settings
☒	☐	For hierarchical and complex designs, identification of the appropriate level for tests and full reporting of outcomes
☒	☐	Estimates of effect sizes (e.g. Cohen's *d*, Pearson's *r*), indicating how they were calculated
☐	☒	Clearly defined error bars<br>*State explicitly what error bars represent (e.g. SD, SE, CI)*

Our web collection on statistics for biologists may be useful.

Software and code

Policy information about availability of computer code

Data collection	No software was used.
Data analysis	"Polo Plus" (LeOra Software, 2002) was used for bioassay data processing.

For manuscripts utilizing custom algorithms or software that are central to the research but not yet described in published literature, software must be made available to editors/reviewers upon request. We strongly encourage code deposition in a community repository (e.g. GitHub). See the Nature Research guidelines for submitting code & software for further information.

Data

Policy information about availability of data

All manuscripts must include a data availability statement. This statement should provide the following information, where applicable:

- Accession codes, unique identifiers, or web links for publicly available datasets
- A list of figures that have associated raw data
- A description of any restrictions on data availability

The authors declare that the data supporting the findings of this study are available within the paper and its Supplementary Information.

Field-specific reporting

Please select the best fit for your research. If you are not sure, read the appropriate sections before making your selection.

☒ Life sciences ☐ Behavioural & social sciences ☐ Ecological, evolutionary & environmental sciences

For a reference copy of the document with all sections, see nature.com/authors/policies/ReportingSummary-flat.pdf

Life sciences study design

All studies must disclose on these points even when the disclosure is negative.

Sample size	For bioassays, hundreds of insects were tested for each chemicals. For biochemical analyses, 3 to 6 biological repeats were conducted.
Data exclusions	No data were excluded from the analyses.
Replication	All attempts at replication were successful.
Randomization	Sample allocation was random.
Blinding	Blinding was not relevant to our study.

Reporting for specific materials, systems and methods

Materials & experimental systems

n/a	Involved in the study	
☒	☐	Unique biological materials
☒	☐	Antibodies
☐	☒	Eukaryotic cell lines
☒	☐	Palaeontology
☒	☐	Animals and other organisms
☒	☐	Human research participants

Methods

n/a	Involved in the study	
☒	☐	ChIP-seq
☒	☐	Flow cytometry
☒	☐	MRI-based neuroimaging

Eukaryotic cell lines

Policy information about cell lines

Cell line source(s)	High Five cells were purchased from Invitrogen Life Technologies.
Authentication	High Five cells are ovarian cells from the insect Trichoplusia ni.
Mycoplasma contamination	The cell lines were tested negative for mycoplasma contamination.
Commonly misidentified lines (See ICLAC register)	Not applicable.
